# WPA Global Guidelines for Telepsychiatry

**DOI:** 10.1192/j.eurpsy.2021.349

**Published:** 2021-08-13

**Authors:** D. Mucic

**Affiliations:** Telepsychiatry, little prince treatment centre, copenhagen v, Denmark

**Keywords:** International collaboration, WPA, Regulative issues, Telepsychiatry Global Guidelines

## Abstract

**Introduction:**

The current pandemic has only confirmed the need for international collaboration and more extended use of telepsychiatry than before. Unfortunately, regulatory constraints and lack of standardization are posing significant barriers to the internationalization of telepsychiatry. A need for global guidelines and service standardizations is of utmost importance in this rapidly growing but not yet well-established field. By mastering telepsychiatry, the professionals also may enable the remote provision of other eMH approaches complementary to well-known, traditional service(s). However, first, one ought to become familiar with the basics of telepsychiatry. Globally standardized telepsychiatric service and uniform regulations are prerequisites for fruitful international cooperation.

**Objectives:**

- to present the main objectives and messages of the WPA Global Guidelines for Telepsychiatry.

**Methods:**

A structured review of the main challenges, innovations, and settings in the first Global Guidelines for Telepsychiatry, published by WPA.

**Results:**

With proper preparation and thoughtful risk management, telepsychiatry can be an invaluable tool for allowing greater access to care. However, certain prerequisites must be fulfilled to achieve the desired goals. These prerequisites are e.g. choice of the technology, settings, patient/provider preferences as well as competencies and skills, all outlined in this document.

**Conclusions:**

This WPA document may pave the way for the development of global regulations in order to break down the barriers of accessibility for both the professionals as well as for the patients worldwide. Further, it may help professionals in setting up a standardized telepsychiatry service(s) in addition to the existing mental health system(s).

**Disclosure:**

I am the author of WPA Global Guidelines for Telepsychiatry but have no financial interest.

